# Acute retinal necrosis: time to consider double dose of Foscarnet in the first 72 hours

**DOI:** 10.22336/rjo.2021.53

**Published:** 2021

**Authors:** Francisco Manuel Hermoso-Fernández, Carmen Gonzalez-Gallardo, María Cruz-Rojo

**Affiliations:** * Department of Ophthalmology, San Cecilio University Hospital, Granada, Spain

**Keywords:** retinal necrosis, viral uveitis, antiviral treatment, varicella zoster, Foscarnet, intravitreal treatment

## Abstract

**Purpose:** To report a case of acute retinal necrosis (ARN) and to emphasize special aspects of the management. Factors that must be considered.

**Methods:** We present the case of an 83-year-old woman examined for acute vision loss in her left eye (LE). Background: diabetes, pseudophakic in her LE; subluxated intraocular lens (IOL) and advanced pseudoexfoliative glaucoma in her right eye (RE). The visual acuity (VA) was hand movements in both eyes. Funduscopic examination revealed vitritis, temporal area of retinal necrosis with peripapillary choroiditis spots and macular haemorrhages in her LE and OCT showed a cystic macular edema.

**Results:** A positive polymerase chain reaction (PCR) test for Varicella Zoster Virus (VZV) in aqueous humor of her LE was found. She underwent intravenous Acyclovir 10 mg per kg every 8 hours. She received two doses of adjunctive intravitreal Foscarnet (2.4 mg/ 0.1 mL) in the first 3 days of treatment (2 days between doses). After 3 days of treatment, she started with intravenous prednisone 60 mg per day. The VA of her LE was 0,8 and the retinal necrosis activity was stationary.

In fundoscopic examination, vitritis and retinal hemorrhages have disappeared. At that moment there were no foci of chorioretinitis or macular edema although retinal ischemia persisted at the inferior nasal level.

**Conclusions:** The role of adjunctive intravitreal antiviral therapy in combination with systemic treatment revealed promising results.

Corticosteroids can be used topically and orally to decrease the severe inflammatory response associated with ARN.

Early treatment is crucial to optimize visual and anatomic outcomes.

## Introduction

Acute retinal necrosis (ARN) is an uncommon viral uveitis syndrome characterized by a diffuse necrotizing retinitis that can lead to devastating visual consequences if not promptly diagnosed and treated. It is caused by human herpes viruses that can affect immunocompetent or immunosuppressed patients of either gender at any age. Vision loss may occur as a result of chronic vitritis, epiretinal membrane, macular ischemia, macular edema, optic neuropathy, and retinal detachment. Early accurate diagnosis of ARN is critical to initiate timely antiviral therapy [**1**]. Analysis of ocular fluid with PCR testing has heralded a sensitivity and specificity greater than 90% in the detection of varicella zoster virus (VZV), herpes simplex virus (HSV), and cytomegalovirus (CMV). Adjunctive intravitreal antiviral therapy in combination with systemic treatment has revealed promising results in recent studies. We present another case of an unexpected resolution after the combination of systemic and intravitreal therapies [**2**]. 

## Case presentation

**Fig. 1:**
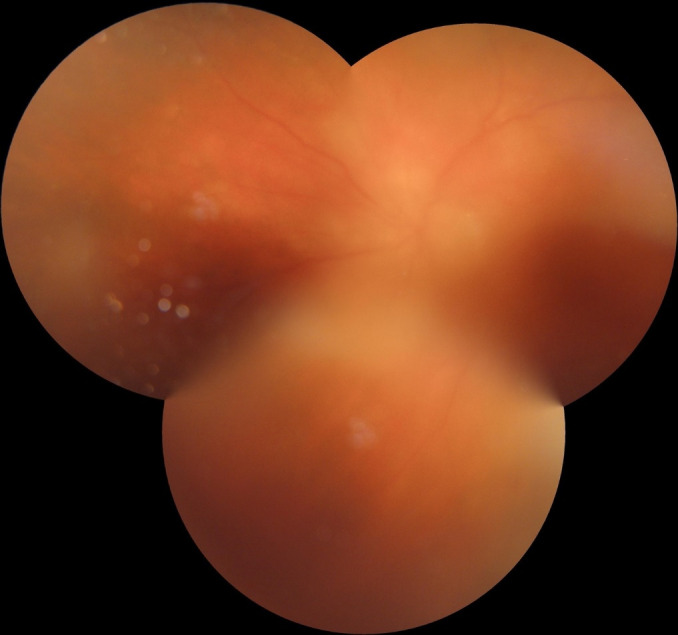
Funduscopic image captured before treatment

We present an 83-year-old woman examined for acute vision loss in her left eye (LE). She was pseudophakic of both eyes (BE), her right intraocular lens (IOL) was subluxated and she also had a pseudoexfoliative glaucoma in her right eye (RE). In the initial exploration, her visual acuity (VA) was hand movements’ recognition in both eyes. Slit-lamp examination showed an acute anterior uveitis with hyperemia and tyndall +++ in her left eye, and subluxated IOL in her right eye. The funduscopic examination revealed vitritis, temporal area of retinal necrosis with peripapillary choroiditis’ spots and macular haemorrhages in her LE (**Fig. 1**).

There were no signs of vitritis or retinitis in her right eye. She was diagnosed with unilateral panuveitis with acute retinal necrosis and she underwent intravenous Aciclovir 10 mg per kg every 8 hours, oral prednisone 60 mg per day, dexamethasone drops every 2 hours and cyclopentolate drops every 8 hours. Despite the treatment, a progression of the retinal ischemia to the inferior area, multiple haemorrhages and papilledema were observed 24 hours later. We decided to treat her with a dose of adjunctive intravitreal Foscarnet (2.4 mg/ 0.1 mL) and at the same time we obtained aqueous humor trough an anterior paracentesis. Afterwards, we obtained a positive polymerase chain reaction (PCR) test for Varicella Zoster Virus (VZV) in aqueous humor.

**Fig. 2:**
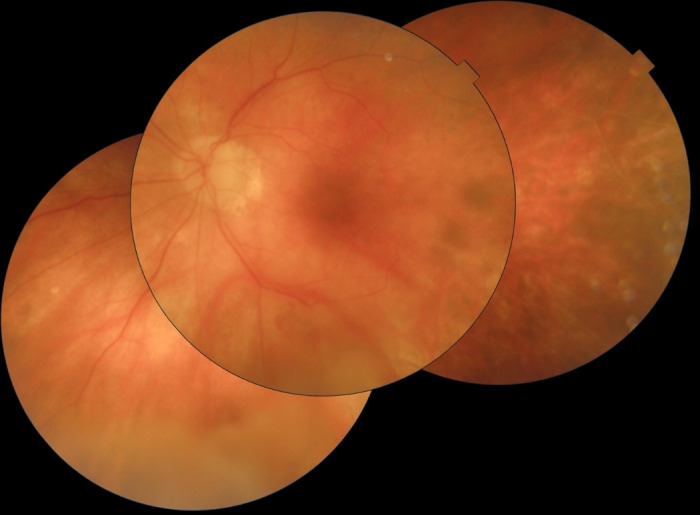
Funduscopic image captured after acute retinal necrosis resolution

The patient referred improvement in her symptoms. Also, we observed a decrease in intraocular inflammation so we decided to inject another dose of adjunctive intravitreal Foscarnet (2.4 mg/ 0.1 mL) 3 days after the first one. After 14 days with intravenous Aciclovir treatment, the VA in her LE was 6/ 20, no inflammation being present in the anterior chamber and the funduscopic examination showed a superior temporal branch retinal artery occlusion and temporary sheathed (superior temporal arteritis) surrounded by many retinal hemorrhages. Papilledema and an extensive area of non-perfused retina were also observed in the nasal periphery (**Fig. 2**). No retinal tears were appreciated. Due to the clinical improvement, it was decided to stop the intravenous treatment and to start with oral Valaciclovir 1 gr every 8 hours and oral prednisone 30 mg per day. 

At the last check, the VA of her LE was 0,8 and the retinal necrosis activity was stationary. No papilledema was present. Many venous occlusions were observed in the periphery and she was under only 1 gr Valaciclovir treatment every 8 hours and oral prednisone 30 mg per day, which was enough to control macular edema.

## Discussion

After a conscientious revision of literature, this is the first reported case of an acute retinal necrosis with such a good resolution. 

Acute retinal necrosis (ARN) represents an intraocular herpetic infection characterized by severe inflammatory symptoms, including progressive retinitis and choroiditis with occlusive vasculitis and papillitis. It has a poor visual prognosis, due to extensive retinal inflammation with necrosis, occlusive vasculitis, frequent development of retinal detachment, and optic disc atrophy [**3**]. The necrotic process has been described to be driven by CD4+T cells, macrophages, polymorphonuclear cells, B cells, and by the inflammatory cytokines TNF-α and IFN-γ [**4**]. Based on that, the corticosteroid oral use could be of great significance to improve inflammatory metrics measures associated with ARN. Consequently, we decided to use oral and topically corticoids despite the fact that larger-scale studies have not demonstrated an improvement in visual or anatomic outcomes with the use of corticosteroids [**5**,**6**].

Historically, intravenous antiviral therapy was the standard of care in treating ARN. However, nowadays, some recent studies suggest that oral valacyclovir achieves comparable outcomes to intravenous acyclovir and can be utilized as the initial induction therapy for ARN [**7**]. In our case, we decided to treat her with IV valaciclovir during the hospitalization and change it to oral during the follow up.

One significant fact that should be pointed out is the role of adjunctive intravitreal antiviral therapy in combination with systemic treatment. Combination therapy had demonstrated to improve visual acuity by two lines or greater, a decreased incidence of progression to severe visual loss and a reduced incidence of retinal detachment. These studies suggested a combination of oral valacyclovir 1,000 mg to 2,000 mg three times daily with serial Foscarnet injections (2.4 mg/ 0.1 mL) every 3 days as induction therapy until disease quiescence is achieved, with maintenance treatment comprised of oral valacyclovir and intravitreal antiviral injections according to the need [**6**]. In our case, we achieved the disease quiescence with the second Foscarnet intravitreal injection.

Any other way, there is insufficient evidence supporting the role of early vitrectomy in the prevention of retinal detachment and severe vision loss [**8**]. Besides, due to good evolution with pharmacological treatment, we decided not to do it. 

On the other hand, not enough evidence was found for prophylactic laser, in order to affect the rate of retinal detachment [**8**], but future studies will need to investigate its role in the prevention of retinal detachment in ARN patients. 

## Conclusion

In conclusion, optimal management of ARN involves prompt treatment with high-dose oral valacyclovir as induction therapy, combined with intravitreal antiviral therapy, but highlighting the importance of prompt treatment while awaiting viral confirmation. In addition, corticosteroid use may improve inflammatory metrics measures associated with ARN. Finally, the evidence regarding prophylactic laser retinopexy and early vitrectomy are not well established at this time. 

**Conflict of Interest statement**

None of the authors has any financial/ conflict of interest to disclose. 

**Informed Consent and Human and Animal Rights statement**

Informed consent has been obtained from all individuals included in this study.

The patient offered her consent for the publication of her identifiable details in relation to the article “Management of acute retinal necrosis: Time to consider double dose of foscarnet in the first 72 hours” in Romanian Journal of Ophthalmology. 

She offered her consent for the publication of her identifiable details, including photograph(s) and/ or videos and/ or case history and/ or details in the text to be published in the above-mentioned journal. She has discussed this consent form with Francisco Manuel Hermoso Fernández, the author of the paper.

**Authorization for the use of human subjects**

Ethical approval: The research related to human use complies with all the relevant national regulations, institutional policies, is in accordance with the tenets of the Helsinki Declaration, and has been approved by the review board of San Cecilio University Hospital, Granada, Spain.

**Acknowledgements**

None.

**Sources of Funding**

Authors have not received founding from any organization related (National Institutes of Health (NIH); Welcome Trust; Howard Hughes Medical Institute (HHMI).

**Disclosures**

The authors declare that they have no interest in relation to this article.

## References

[1] Schoenberger SD, Kim SJ, Thorne JE, Mruthyunjaya P, Yeh S, Bakri SJ (2017). Diagnosis and treatment of acute retinal necrosis: a report by the American Academy of Ophthalmology. Ophthalmology.

[2] Li AL, Fine HF, Shantha JG, Yeh S (2019). Update on the management of acute retinal necrosis. Ophthalmic Surgery, Lasers and Imaging Retina.

[3] De Visser L, de Boer JH, Rijkers GT, Wiertz K, van den Ham H-J, de Boer R (2017). Cytokines and chemokines involved in acute retinal necrosis. Investigative Ophthalmology & Visual Science.

[4] Archin NM, Atherton SS (2002). Infiltration of T-lymphocytes in the brain after anterior chamber inoculation of a neurovirulent and neuroinvasive strain of HSV-1. Journal of Neuroimmunology.

[5] Flaxel CJ, Yeh S, Lauer AK (2013). Combination systemic and intravitreal antiviral therapy in the management of acute retinal necrosis syndrome (an American Ophthalmological Society thesis). Transactions of the American Ophthalmological Society.

[6] Yeh S, Suhler EB, Smith JR, Bruce B, Fahle G, Bailey ST (2014). Combination systemic and intravitreal antiviral therapy in the management of acute retinal necrosis syndrome. Ophthalmic Surgery, Lasers and Imaging Retina.

[7] Tibbetts MD, Shah CP, Young LH, Duker JS, Maguire JI, Morley MG (2010). Treatment of acute retinal necrosis. Ophthalmology.

[8] Risseeuw S, de Boer JH, Ninette H, van Leeuwen R (2019). Risk of rhegmatogenous retinal detachment in acute retinal necrosis with and without prophylactic intervention. American Journal of Ophthalmology.

